# Supplementary material for: POLYRETINA restores light responses in vivo in blind Göttingen minipigs
Source: Nat Commun. 2022 Jun 27;13:3678. doi: 10.1038/s41467-022-31180-z (PMC9237028; doi:10.1038/s41467-022-31180-z)
Supplement: Supplementary file 1 — Supplementary Information [file 41467_2022_31180_MOESM1_ESM.pdf]

Supplementary information

POLYRETINA restores light responses in-vivo in blind Göttingen minipigs

Paola Vagni[1,°], Marta Jole Ildelfonsa Airaghi Leccardi[1,°], Charles-Henri Vila[1,°], Elodie Geneviève Zollinger[1], Golnaz Sherafatipour[1], Thomas J. Wolfensberger[2] and Diego Ghezzi[1,*]

[1] Medtronic Chair in Neuroengineering, Center for Neuroprosthetics and Institute of Bioengineering, School of Engineering, École Polytechnique Fédérale de Lausanne, Switzerland

[2] Department of Ophthalmology, University of Lausanne, Hôpital Ophtalmique Jules-Gonin, Fondation Asile des Aveugles, Switzerland

° These authors contributed equally to this work

[*]Correspondence and requests for materials should be addressed to Diego Ghezzi (diego.ghezzi@epfl.ch)

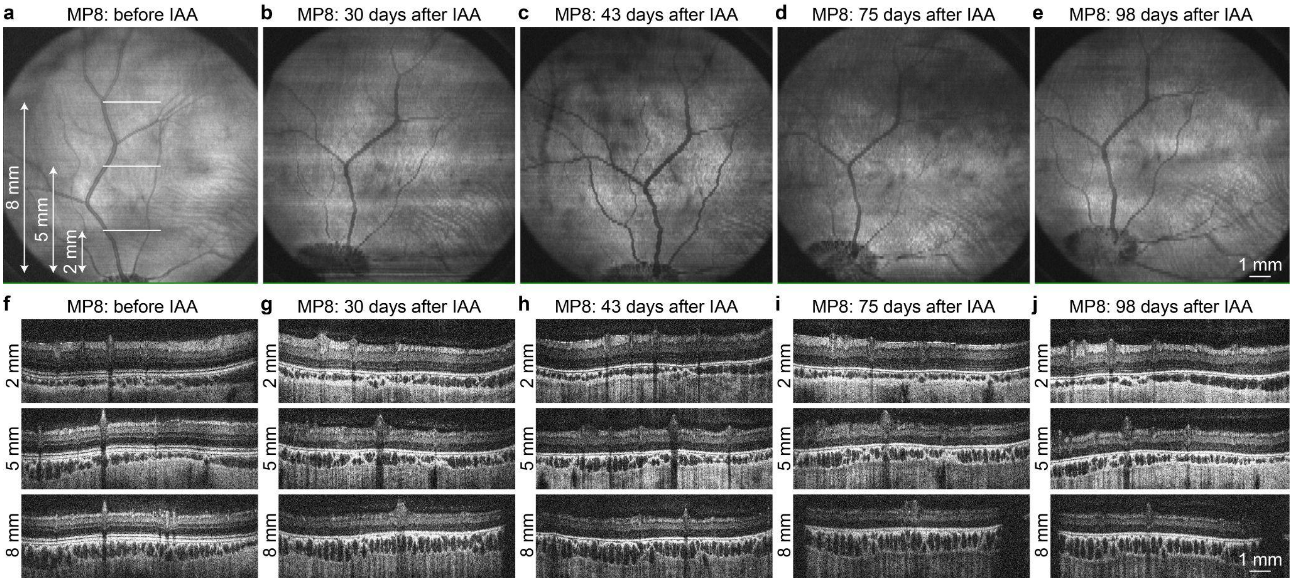

Supplementary Figure 1 | Longitudinal SD-OCT analysis in an IAA-treated Göttingen minipig. a-e, Fundus images of one eye before IAA administration (**a**) and at several time points after IAA administration: 30 days (**b**), 43 days (**c**), 75 days (**d**) and 98 days (**e**). The white lines in panel **a** show the distances from the optic disc at which SD-OCT images were taken: 2, 5 and 8 mm. **f-j**, SD-OCT images in the same eye before IAA administration (**f**) and at matching time points after IAA administration: 30 days (**g**), 43 days (**h**), 75 days (**i**) and 98 days (**j**). Images are from MP8.

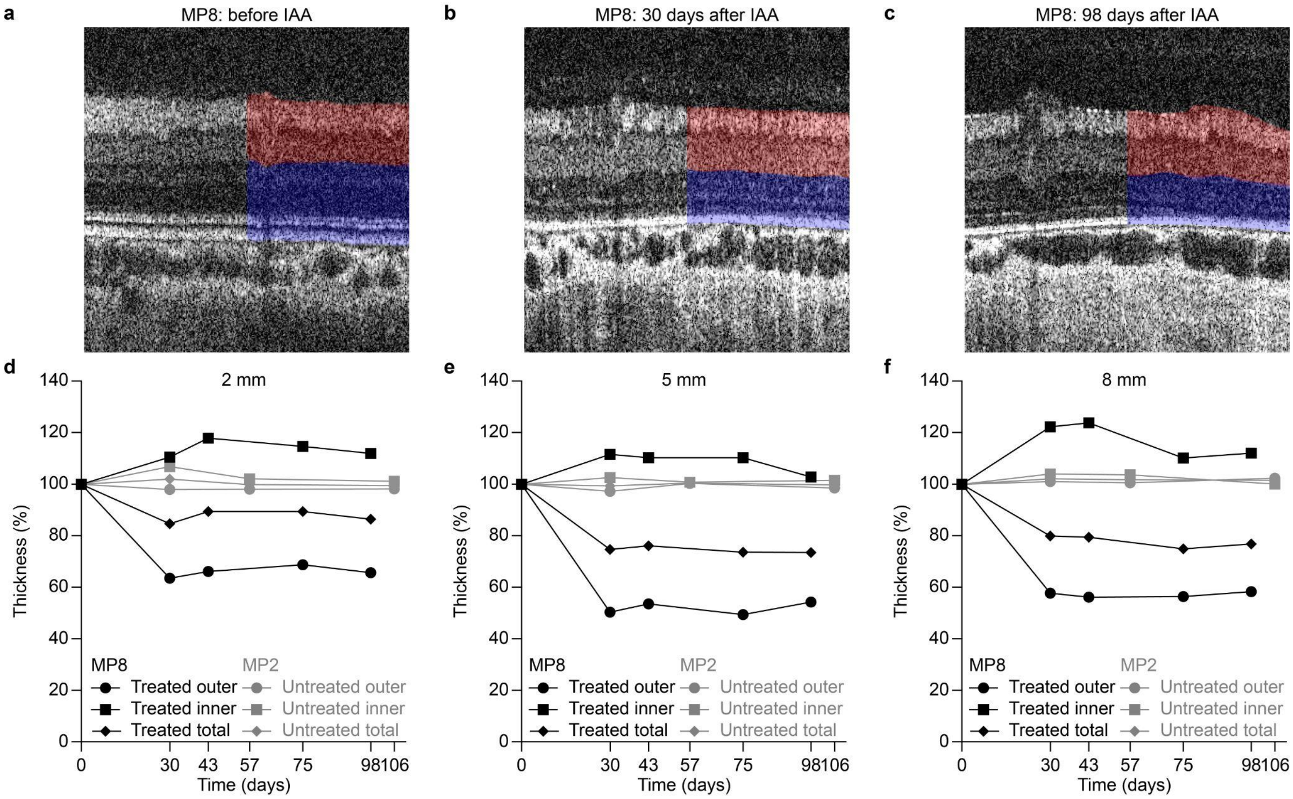

Supplementary Figure 2 | Longitudinal SD-OCT measures in IAA-treated and untreated Göttingen minipigs. **a-c**, Identification in SD-OCT images of the inner retina (red shading) and the outer retina (blue shading) of one eye before IAA administration (**a**), 30 days after IAA administration (**b**) and 98 days after IAA administration (**c**). **d-f**, Quantification of the total retina, the outer retina and the inner retina thicknesses over time for one IAA-treated eye (black) and one untreated eye (grey) at the three distances from the optic disc: 2 mm (**d**), 5 mm (**e**) and 8 mm (**f**). IAA was administered on day 0 after the experiment. Images in panels **a-c** are from MP8. Data in panels **d-f** are from MP8 (IAA-treated) and MP2 (untreated). Source data are provided as a Source Data file.

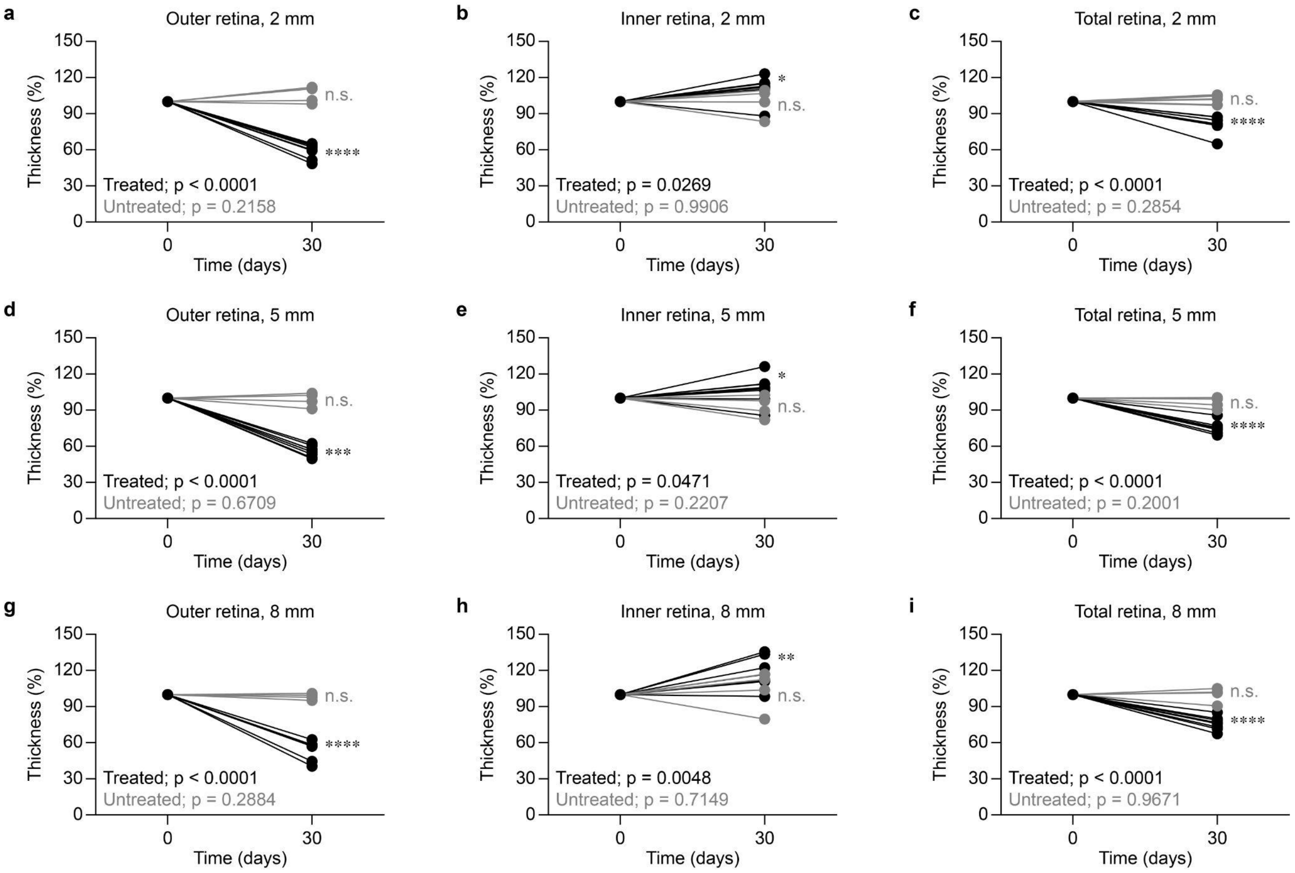

Supplementary Figure 3 | SD-OCT cumulative measures in IAA-treated and untreated Göttingen minipigs. Comparison of the outer (**a**, **d**, and **g**), the inner (**b**, **e**, and **h**) and the total (**c**, **f**, and **i**) retinal thicknesses in IAA-treated minipigs (n = 10 eyes from N = 5 minipigs) before and 1 month after IAA administration and in untreated minipigs (n = 4 eyes from N = 2 minipigs) at matching time points. Measures were obtained at three distances from the optic disc: 2 mm (**a**, **b**, and **c**), 5 mm (**d**, **e**, and **f**), and 8 mm (**g**, **h**, and **i**). IAA was administered on day 0 at the end of the experiment. The p-values in each figure are the results of two-tailed paired t-tests. In each panel, p-values are also reported as: n.s. not significant, * $p < 0.05$, ** $p < 0.01$, *** $p < 0.001$, and **** $p < 0.0001$. Data are from MP4-8 (IAA-treated) and MP1-2 (untreated). Source data are provided as a Source Data file.

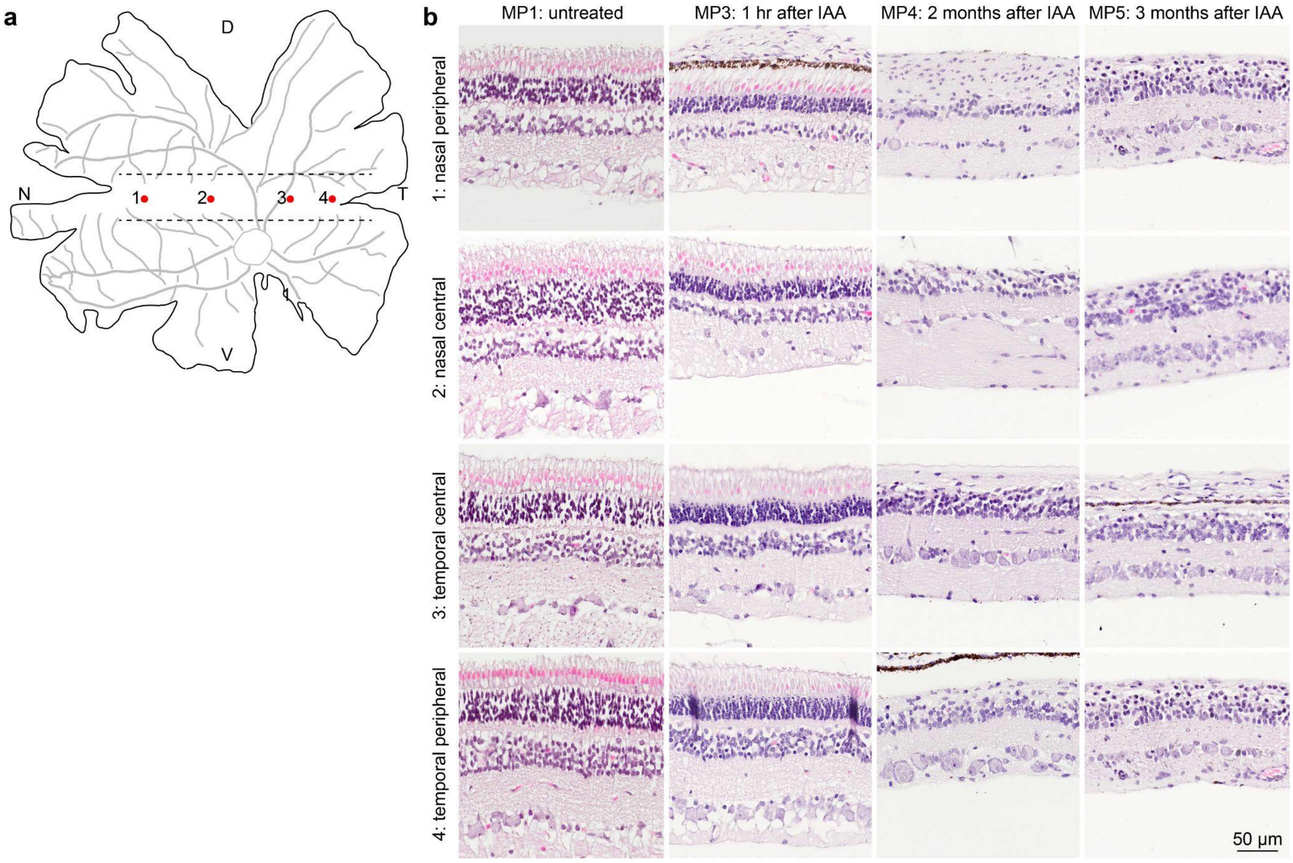

Supplementary Figure 4 | H&E staining at the level of the area centralis. **a**, Drawing of a flattened retina. The dashed lines delimit the area centralis, while the red circles indicate the points corresponding to the images in panel **b** (1: nasal peripheral; 2: nasal central; 3: temporal central; 4: temporal peripheral). D: dorsal; T: temporal; V: ventral; N: nasal. **b**, Images of the stained retinas at various time points (untreated control, 1 hour after IAA administration, 2 months after IAA administration and 3 months after IAA administration) and locations. The images at the same time point are from the same eye. The images at different time points are from different animals (n = 1 eye from N = 1 minipig per time point). Images are from MP1 and MP3-5.

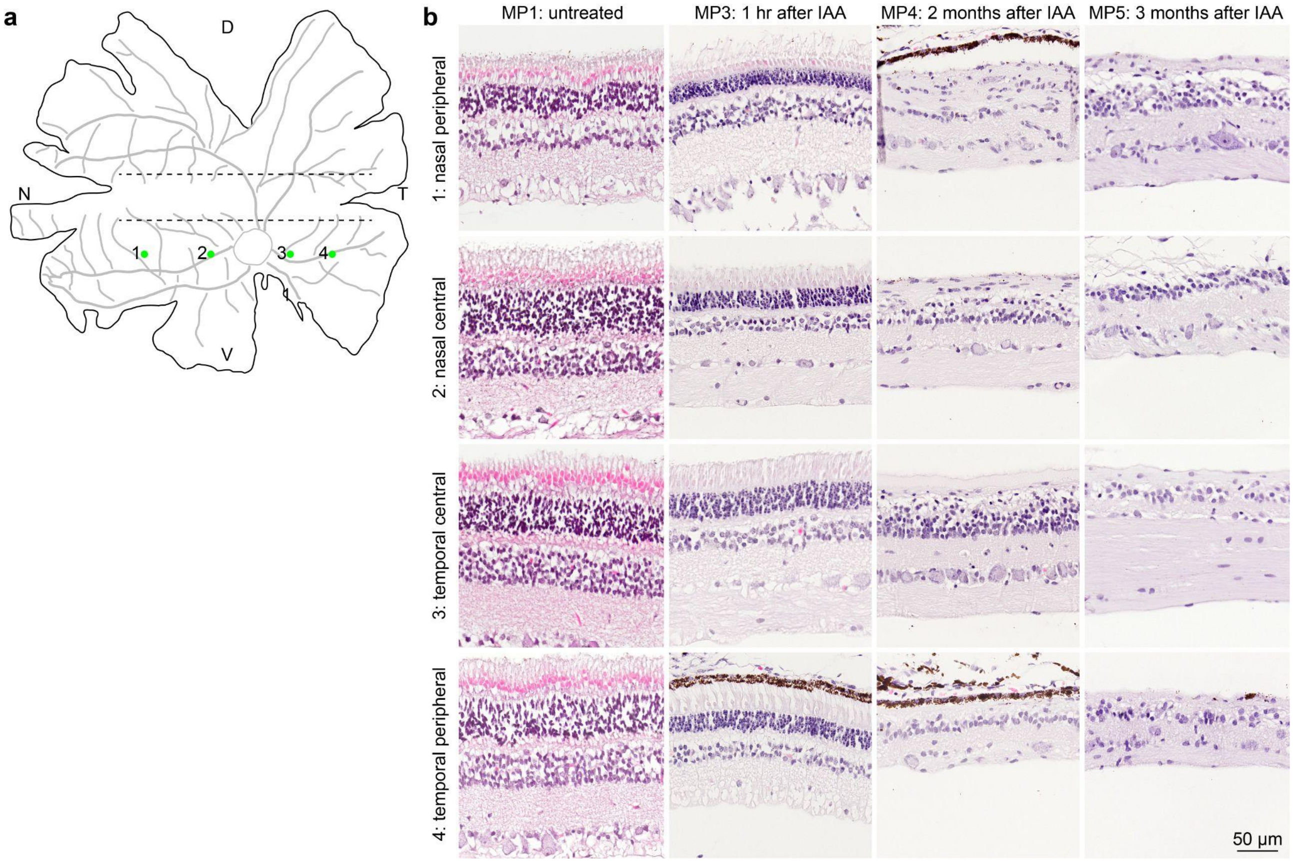

Supplementary Figure 5 | H&E staining at the level of the optic disc. **a**, Drawing of a flattened retina. The dashed lines delimit the area centralis, while the green circles indicate the points corresponding to the images in panel **b** (1: nasal peripheral; 2: nasal central; 3: temporal central; 4: temporal peripheral). D: dorsal; T: temporal; V: ventral; N: nasal. **b**, Images of the stained retinas at various time points (untreated control, 1 hour after IAA administration, 2 months after IAA administration and 3 months after IAA administration) and locations. The images at the same time point are from the same eye. The images at different time points are from different animals (n = 1 eye from N = 1 minipig per time point). Images are from MP1 and MP3-5.

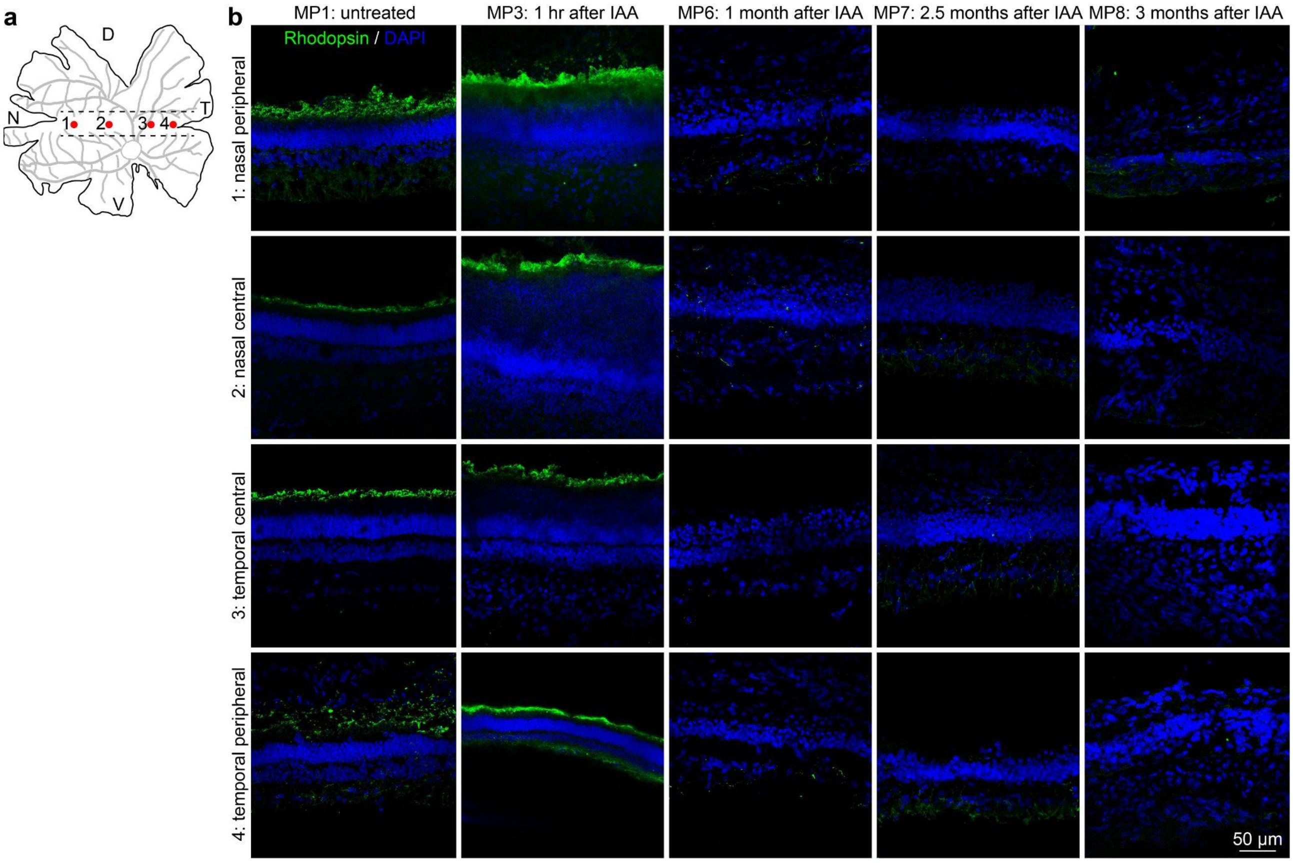

Supplementary Figure 6 | IHC staining against rhodopsin at the level of the area centralis. **a**, Drawing of a flattened retina. The dashed lines delimit the area centralis, while the red circles indicate the points corresponding to the images in panel **b** (1: nasal peripheral; 2: nasal central; 3: temporal central; 4: temporal peripheral). D: dorsal; T: temporal; V: ventral; N: nasal. **b**, Images of the stained retinas at various time points (untreated control, 1 hour after IAA administration, 1 month after IAA administration, 2.5 months and 3 months after IAA administration) and locations. The images at the same time point are from the same eye. The images at different time points are from different animals (n = 1 eye from N = 1 minipig per time point). Images are from MP1, MP3, and MP6-8.

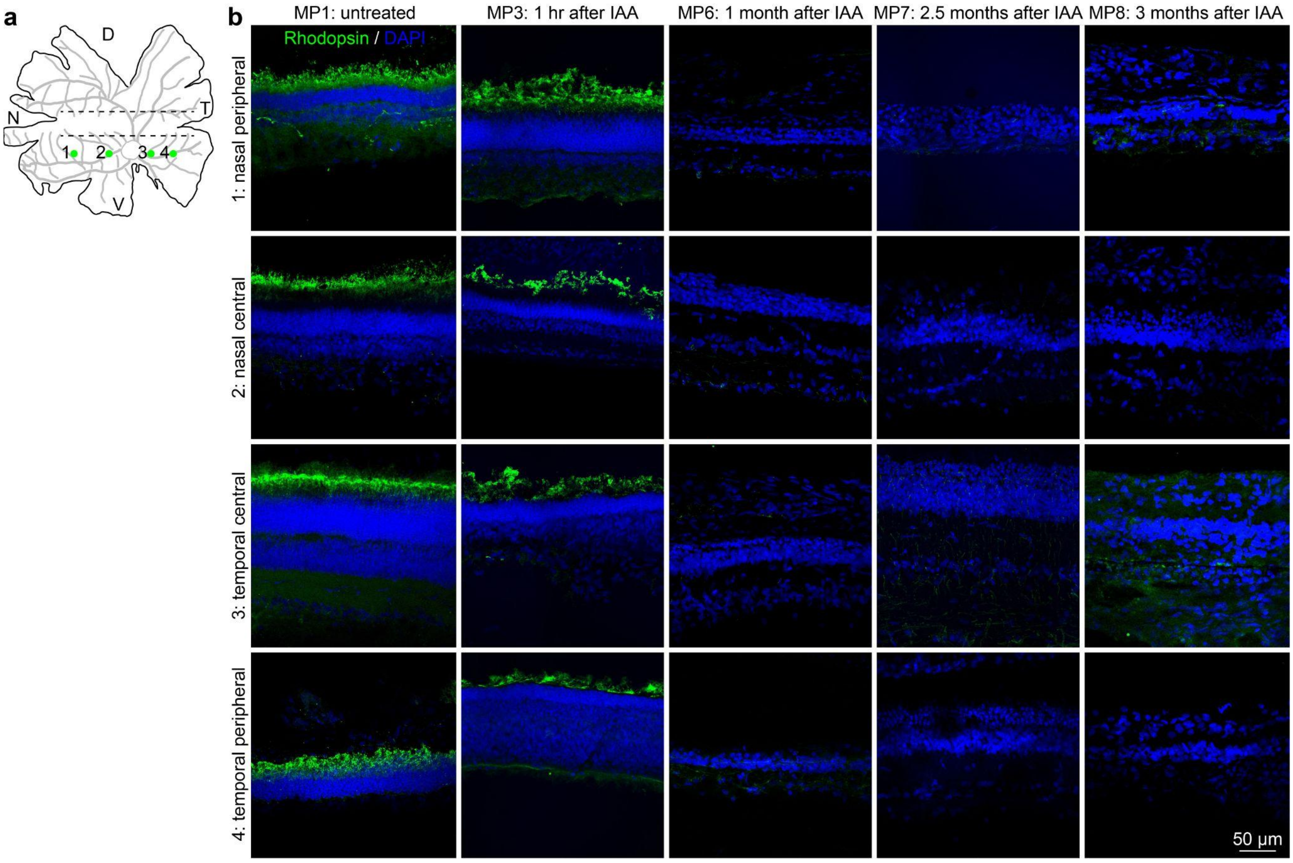

Supplementary Figure 7 | IHC staining against rhodopsin at the level of the optic disc. a, Drawing of a flattened retina. The dashed lines delimit the area centralis, while the green circles indicate the points corresponding to the images in panel **b** (1: nasal peripheral; 2: nasal central; 3: temporal central; 4: temporal peripheral) . D: dorsal; T: temporal; V: ventral; N: nasal. **b**, Images of the stained retinas at various time points (untreated control, 1 hour after IAA administration, 1 month after IAA administration, 2.5 months and 3 months after IAA administration) and locations. The images at the same time point are from the same eye. The images at different time points are from different animals (n = 1 eye from N = 1 minipig per time point). Images are from MP1, MP3, and MP6-8.

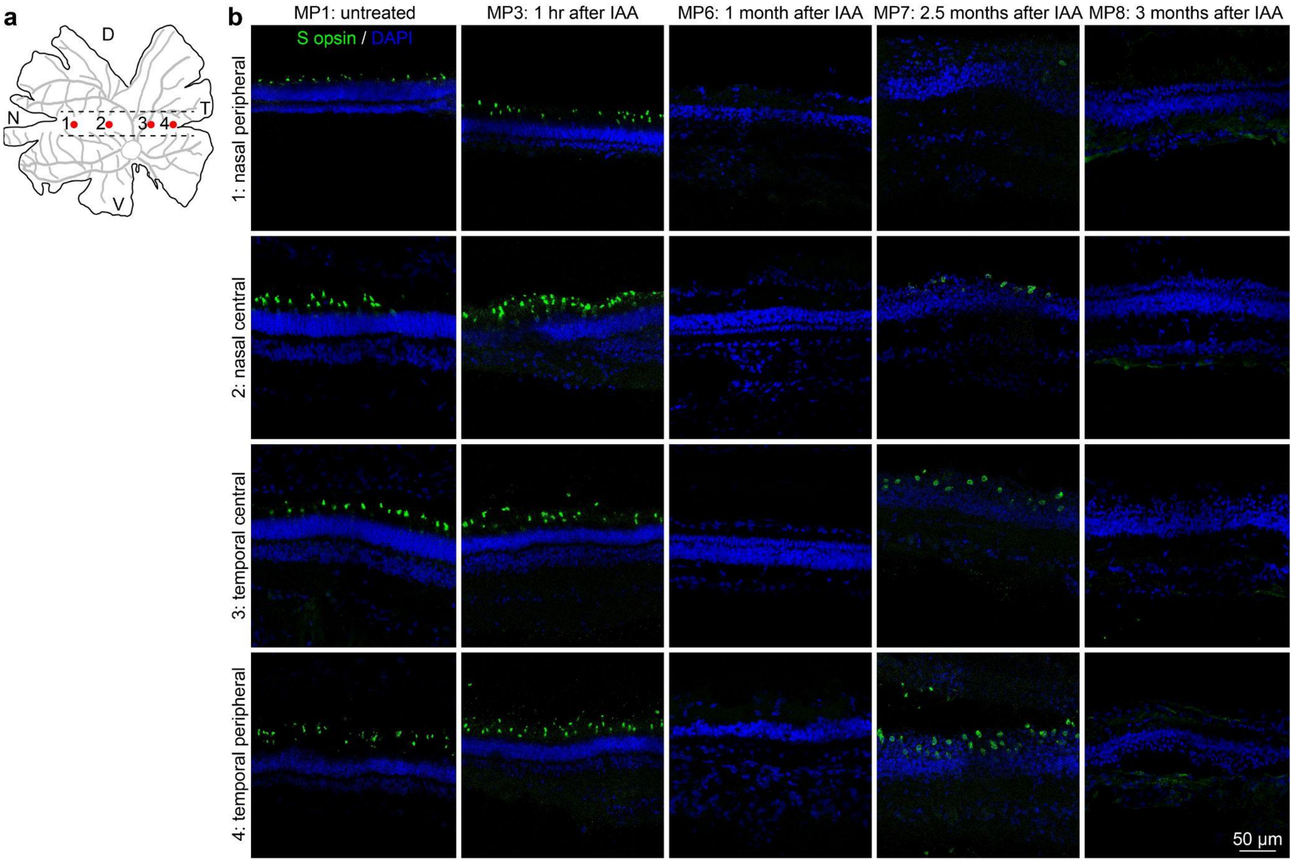

Supplementary Figure 8 | IHC staining against S opsin at the level of the area centralis. **a**, Drawing of a flattened retina. The dashed lines delimit the area centralis, while the red circles indicate the points corresponding to the images in panel **b** (1: nasal peripheral; 2: nasal central; 3: temporal central; 4: temporal peripheral). D: dorsal; T: temporal; V: ventral; N: nasal. **b**, Images of the stained retinas at various time points (untreated control, 1 hour after IAA administration, 1 month after IAA administration, 2.5 months and 3 months after IAA administration) and locations. The images at the same time point are from the same eye. The images at different time points are from different animals (n = 1 eye from N = 1 minipig per time point). Images are from MP1, MP3, and MP6-8.

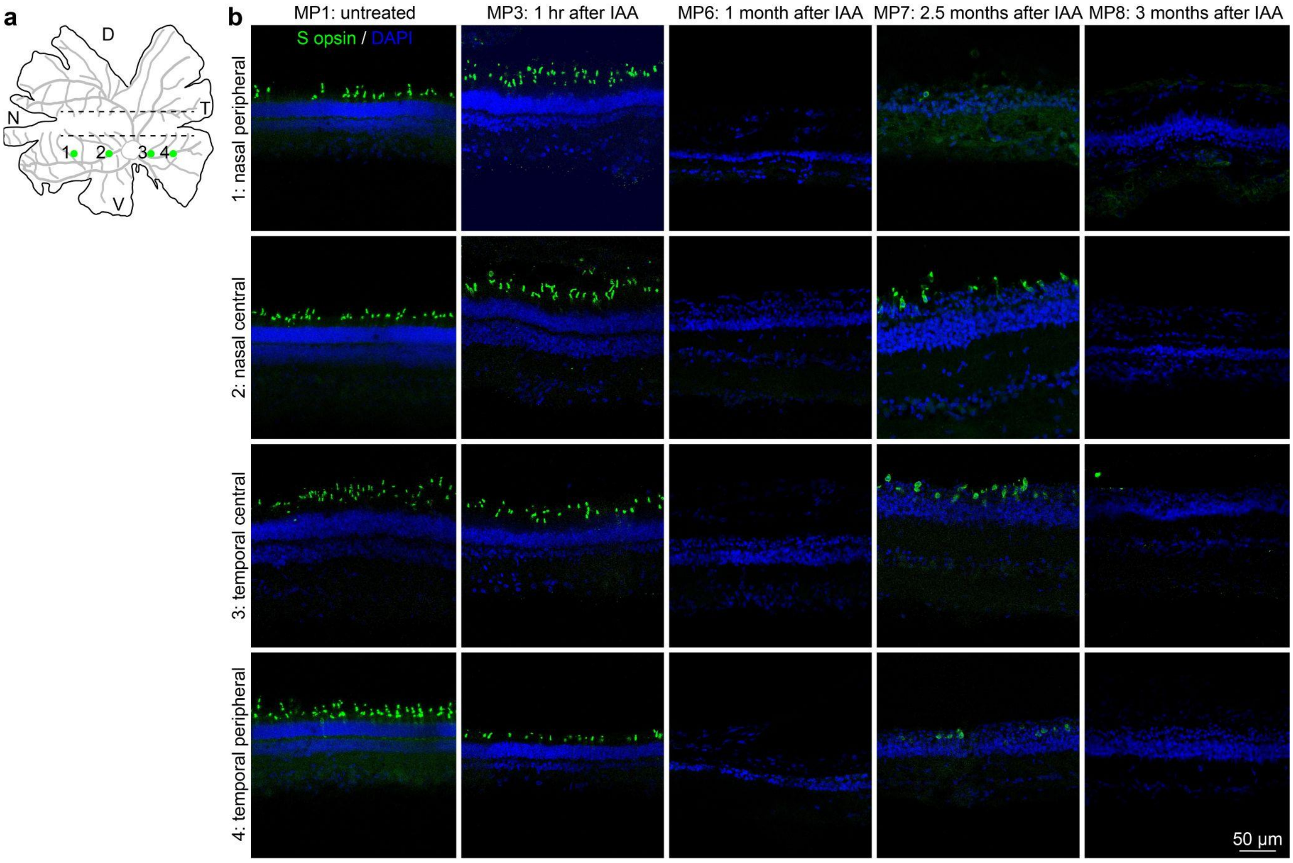

Supplementary Figure 9 | IHC staining against S opsin at the level of the optic disc. **a**, Drawing of a flattened retina. The dashed lines delimit the area centralis, while the green circles indicate the points corresponding to the images in panel **b** (1: nasal peripheral; 2: nasal central; 3: temporal central; 4: temporal peripheral). D: dorsal; T: temporal; V: ventral; N: nasal. **b**, Images of the stained retinas at various time points (untreated control, 1 hour after IAA administration, 1 month after IAA administration, 2.5 months and 3 months after IAA administration) and locations. The images at the same time point are from the same eye. The images at different time points are from different animals (n = 1 eye from N = 1 minipig per time point). Images are from MP1, MP3, and MP6-8.

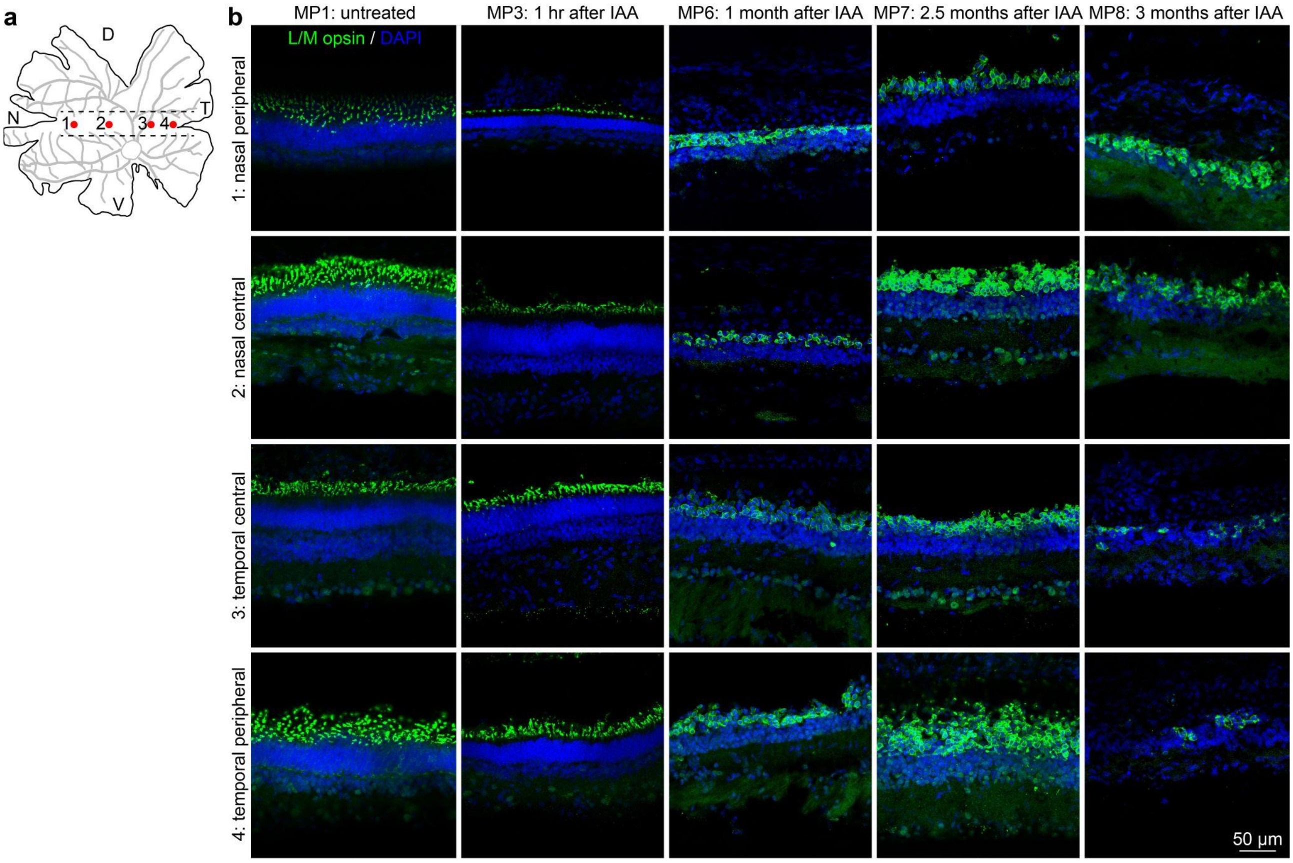

Supplementary Figure 10 | IHC staining against L/M opsin at the level of the area centralis. **a**, Drawing of a flattened retina. The dashed lines delimit the area centralis, while the red circles indicate the points corresponding to the images in panel **b** (1: nasal peripheral; 2: nasal central; 3: temporal central; 4: temporal peripheral). D: dorsal; T: temporal; V: ventral; N: nasal. **b**, Images of the stained retinas at various time points (untreated control, 1 hour after IAA administration, 1 month after IAA administration, 2.5 months and 3 months after IAA administration) and locations. The images at the same time point are from the same eye. The images at different time points are from different animals (n = 1 eye from N = 1 minipig per time point). Images are from MP1, MP3, and MP6-8.

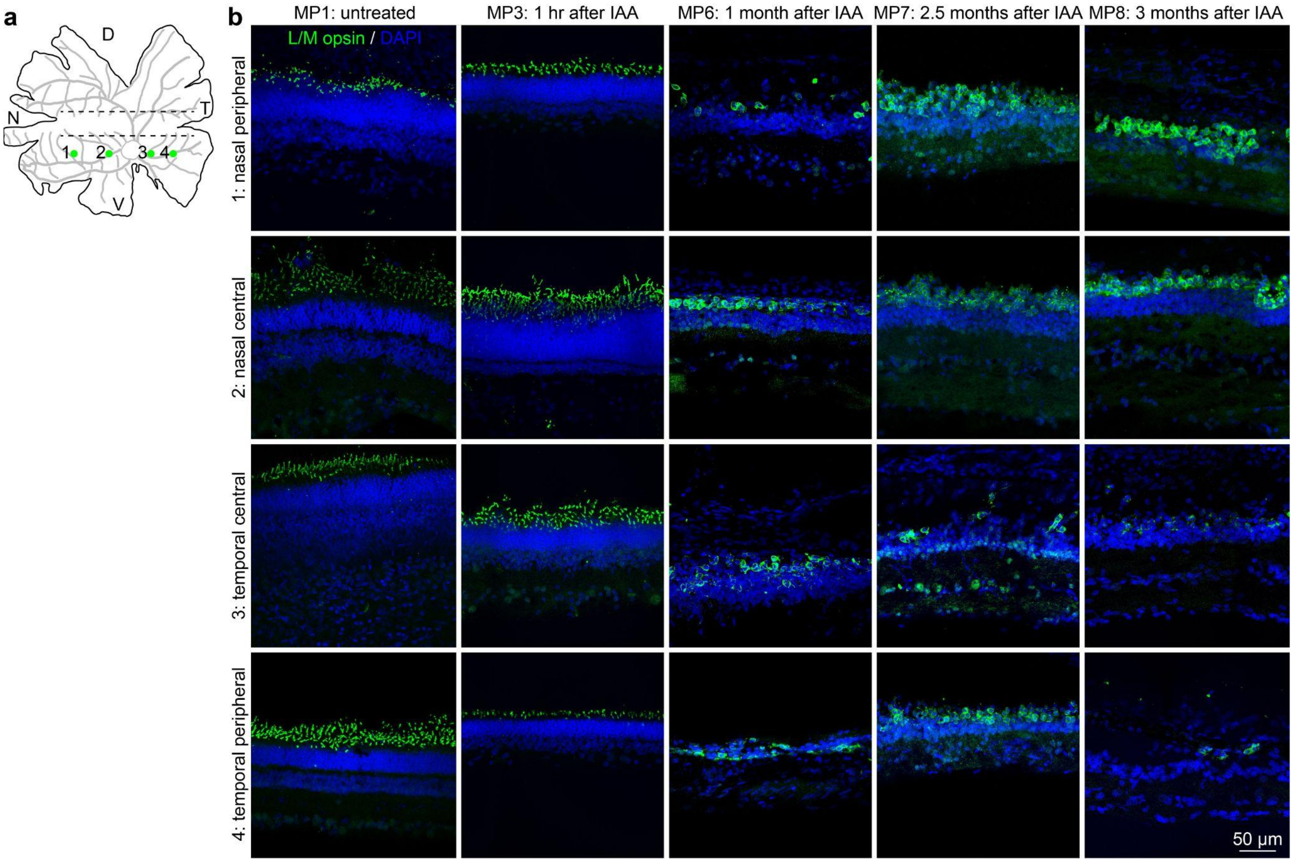

Supplementary Figure 11 | IHC staining against L/M opsin at the level of the optic disc. **a**, Drawing of a flattened retina. The dashed lines delimit the area centralis, while the green circles indicate the points corresponding to the images in panel **b** (1: nasal peripheral; 2: nasal central; 3: temporal central; 4: temporal peripheral). D: dorsal; T: temporal; V: ventral; N: nasal. **b**, Images of the stained retinas at various time points (untreated control, 1 hour after IAA administration, 1 month after IAA administration, 2.5 months and 3 months after IAA administration) and locations. The images at the same time point are from the same eye. The images at different time points are from different animals (n = 1 eye from N = 1 minipig per time point). Images are from MP1, MP3, and MP6-8.

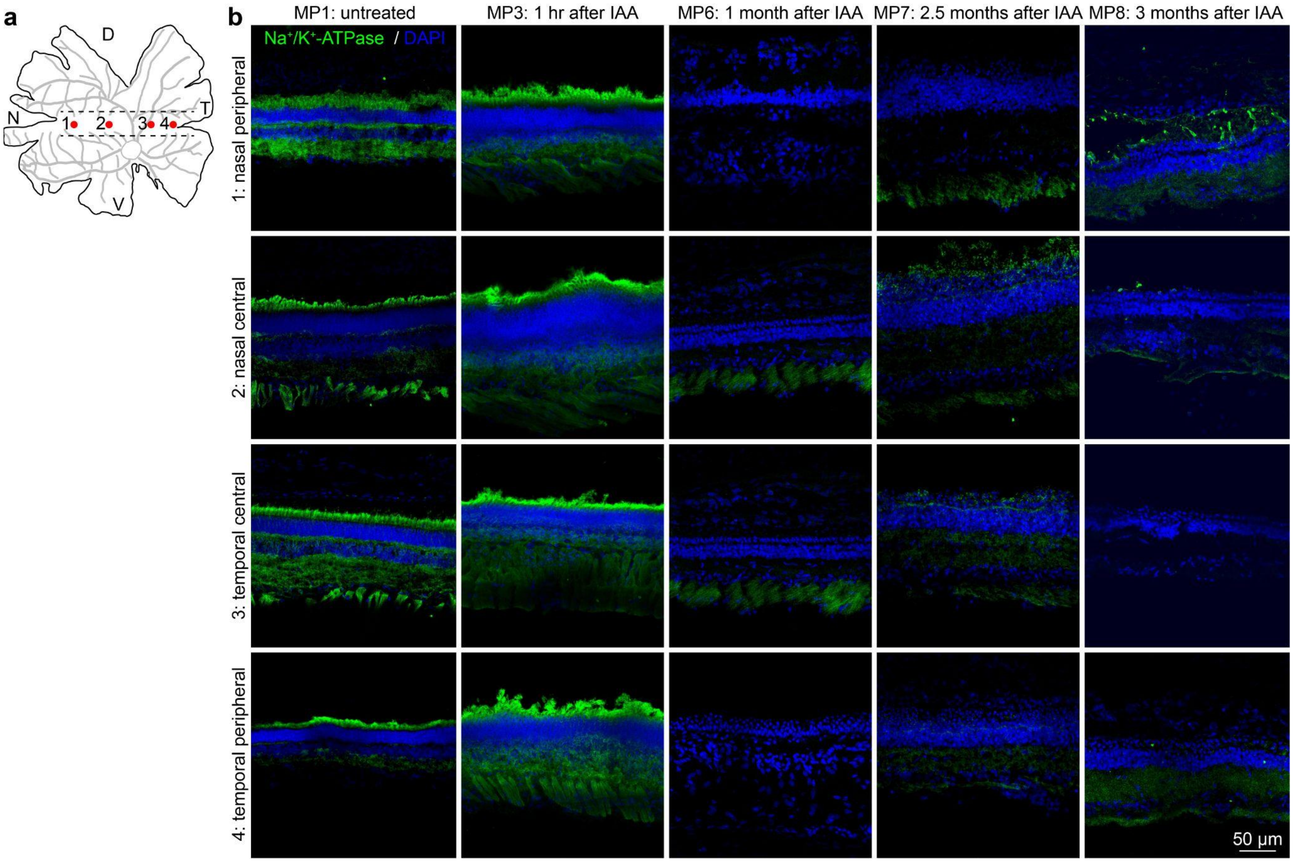

Supplementary Figure 12 | IHC staining against Na^+/K^+-ATPase at the level of the area centralis. **a**, Drawing of a flattened retina. The dashed lines delimit the area centralis, while the red circles indicate the points corresponding to the images in panel **b** (1: nasal peripheral; 2: nasal central; 3: temporal central; 4: temporal peripheral). D: dorsal; T: temporal; V: ventral; N: nasal. **b**, Images of the stained retinas at various time points (untreated control, 1 hour after IAA administration, 1 month after IAA administration, 2.5 months and 3 months after IAA administration) and locations. The images at the same time point are from the same eye. The images at different time points are from different animals (n = 1 eye from N = 1 minipig per time point). Images are from MP1, MP3, and MP6-8.

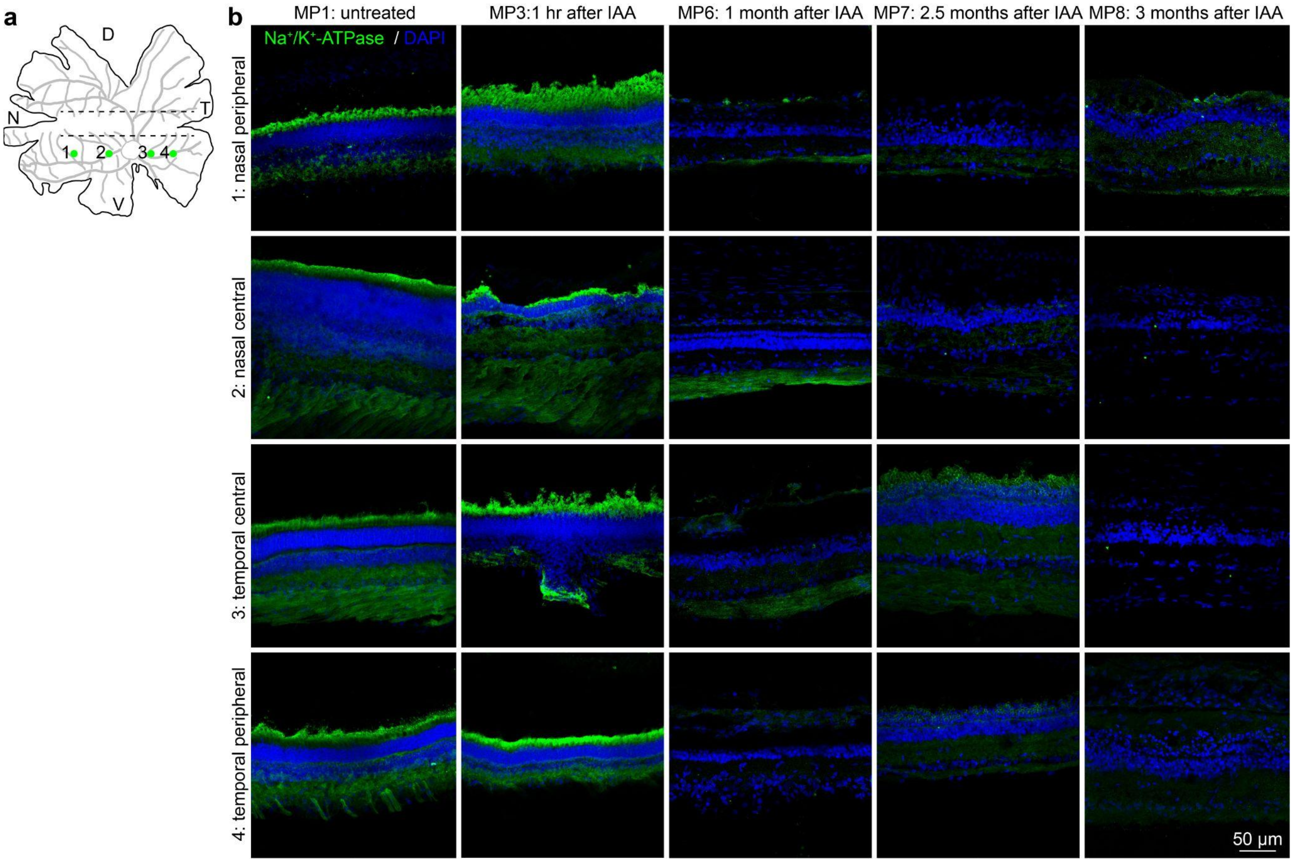

Supplementary Figure 13 | IHC staining against Na^{+}/K^{+}-ATPase at the level of the optic disc. **a**, Drawing of a flattened retina. The dashed lines delimit the area centralis, while the green circles indicate the points corresponding to the images in panel **b** (1: nasal peripheral; 2: nasal central; 3: temporal central; 4: temporal peripheral). D: dorsal; T: temporal; V: ventral; N: nasal. **b**, Images of the stained retinas at various time points (untreated control, 1 hour after IAA administration, 1 month after IAA administration, 2.5 months and 4 months after IAA administration) and locations. The images at the same time point are from the same eye. The images at different time points are from different animals (n = 1 eye from N = 1 minipig per time point). Images are from MP1, MP3, and MP6-8.

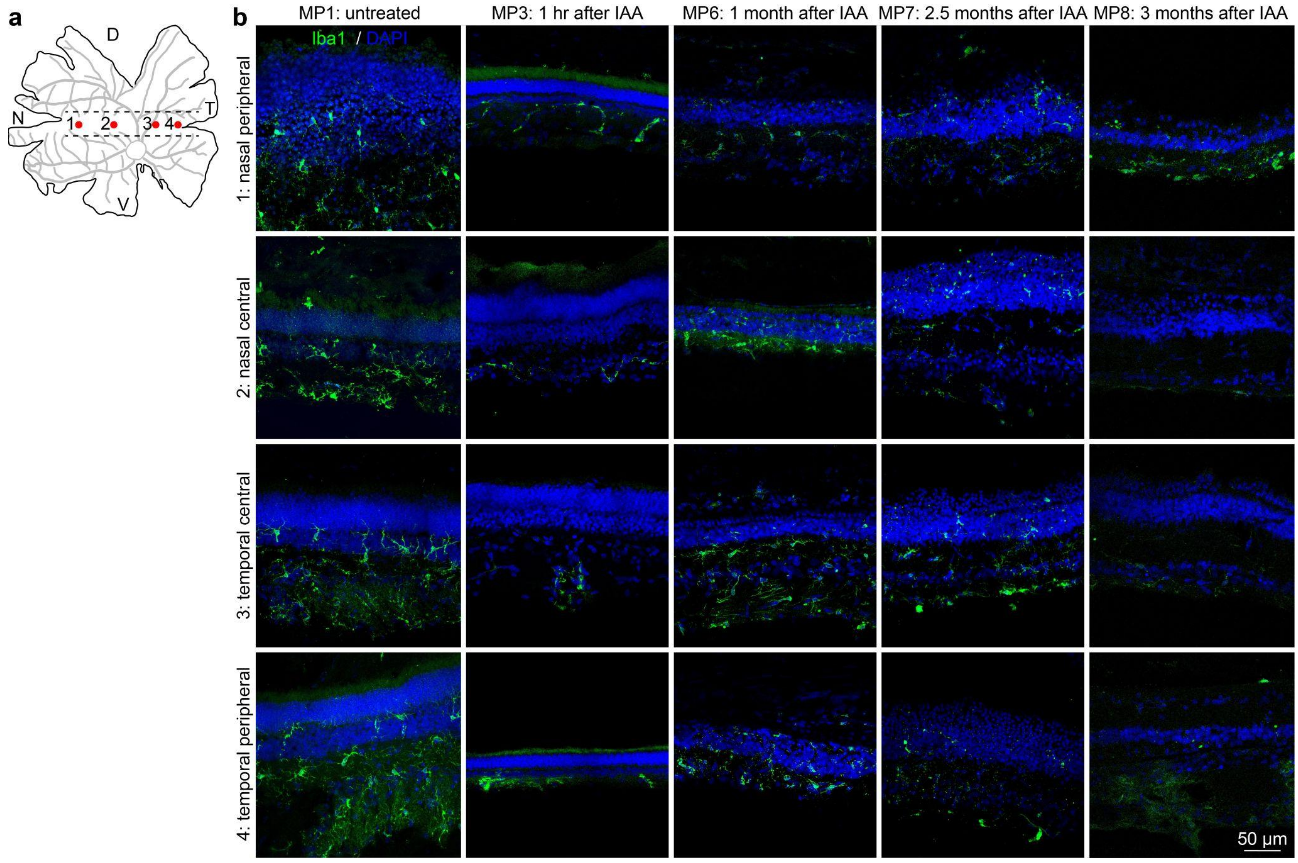

Supplementary Figure 14 | IHC staining against Iba1 at the level of the area centralis. **a**, Drawing of a flattened retina. The dashed lines delimit the area centralis, while the red circles indicate the points corresponding to the images in panel **b** (1: nasal peripheral; 2: nasal central; 3: temporal central; 4: temporal peripheral). D: dorsal; T: temporal; V: ventral; N: nasal. **b**, Images of the stained retinas at various time points (untreated control, 1 hour after IAA administration, 1 month after IAA administration, 2.5 months and 3 months after IAA administration) and locations. The images at the same time point are from the same eye. The images at different time points are from different animals (n = 1 eye from N = 1 minipig per time point). Images are from MP1, MP3, and MP6-8.

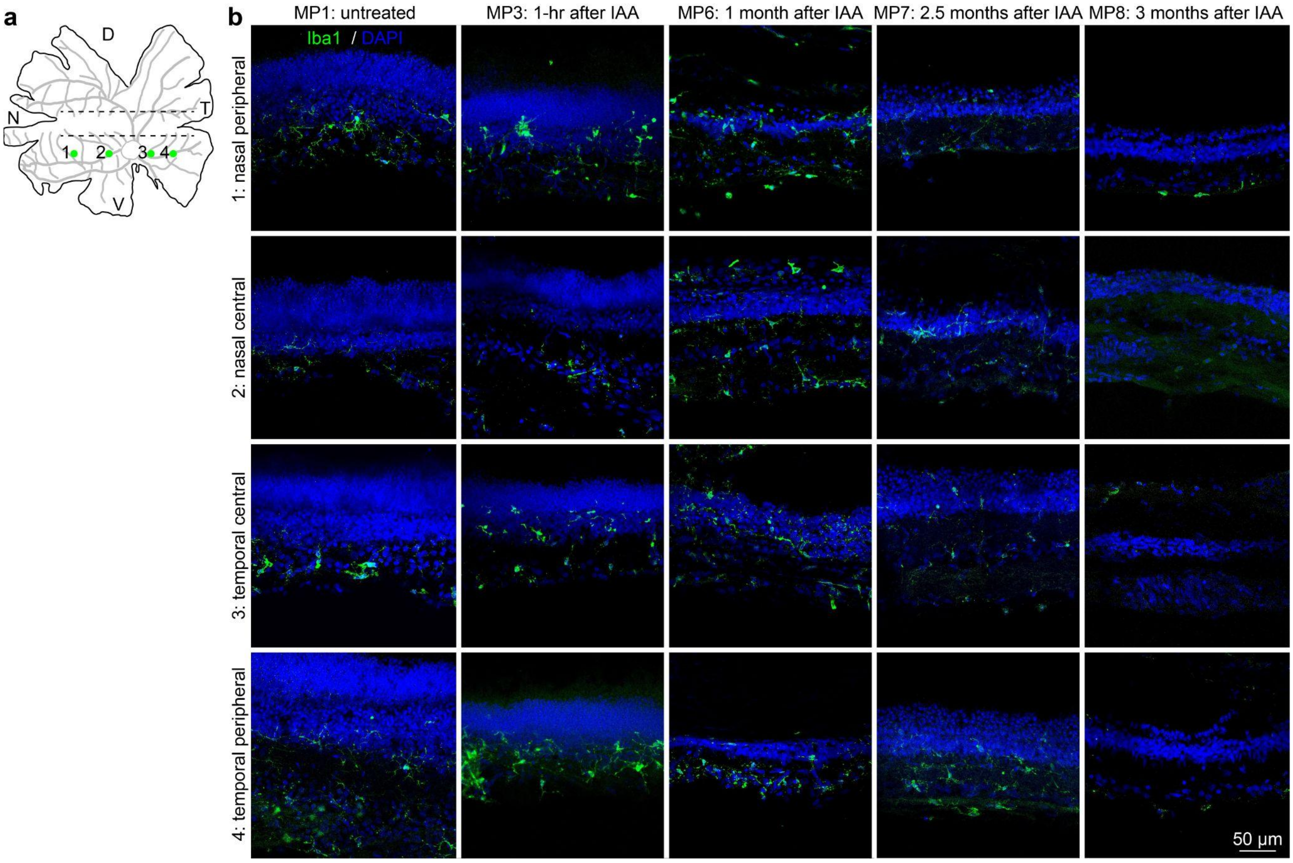

Supplementary Figure 15 | IHC staining against Iba1 at the level of the optic disc. **a**, Drawing of a flattened retina. The dashed lines delimit the area centralis, while the green circles indicate the points corresponding to the images in panel **b** (1: nasal peripheral; 2: nasal central; 3: temporal central; 4: temporal peripheral). D: dorsal; T: temporal; V: ventral; N: nasal. **b**, Images of the stained retinas at various time points (untreated control, 1 hour after IAA administration, 1 month after IAA administration, 2.5 months and 4 months after IAA administration) and locations. The images at the same time point are from the same eye. The images at different time points are from different animals (n = 1 eye from N = 1 minipig per time point). Images are from MP1, MP3, and MP6-8.

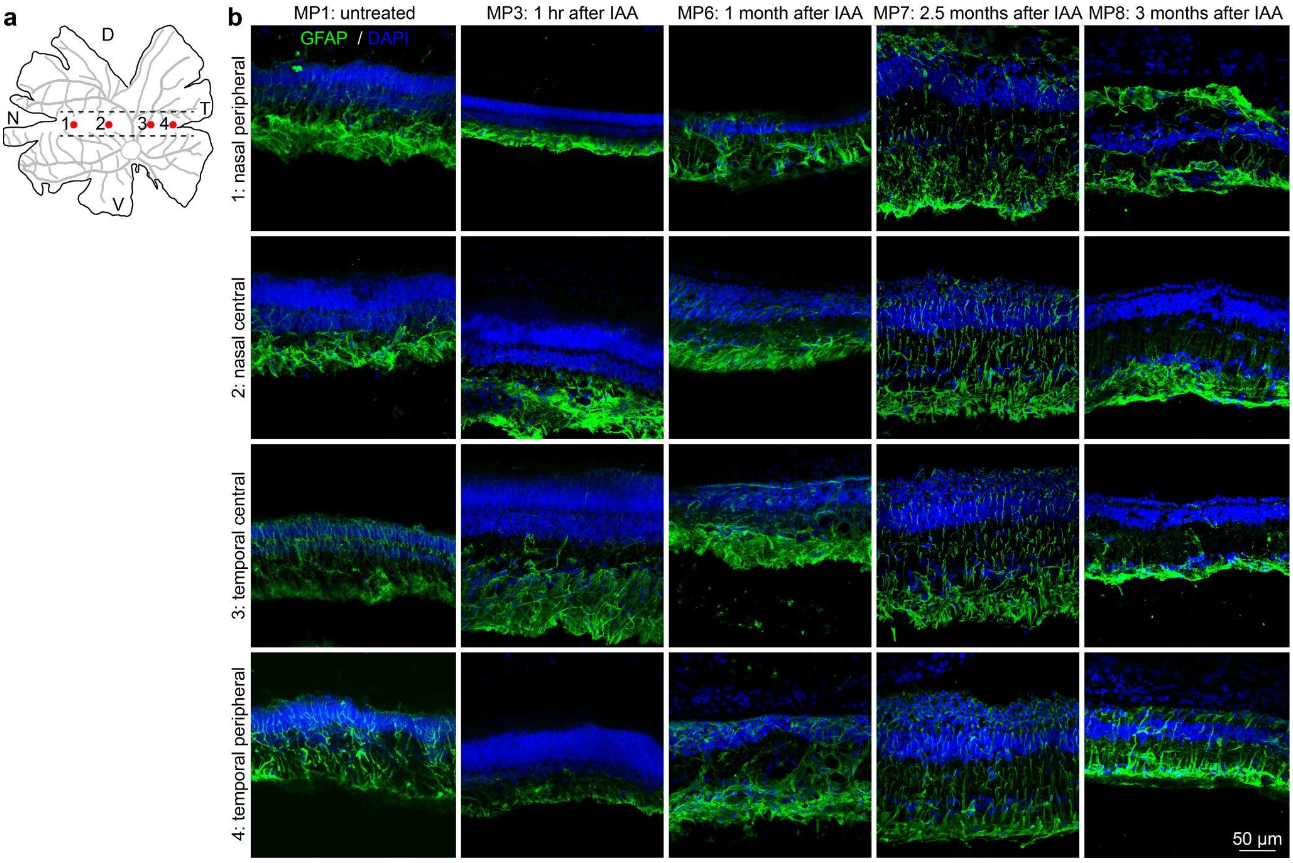

Supplementary Figure 16 | IHC staining against GFAP at the level of the area centralis. **a**, Drawing of a flattened retina. The dashed lines delimit the area centralis, while the red circles indicate the points corresponding to the images in panel **b** (1: nasal peripheral; 2: nasal central; 3: temporal central; 4: temporal peripheral). D: dorsal; T: temporal; V: ventral; N: nasal. **b**, Images of the stained retinas at various time points (untreated control, 1 hour after IAA administration, 1 month after IAA administration, 2.5 months and 3 months after IAA administration) and locations. The images at the same time point are from the same eye. The images at different time points are from different animals (n = 1 eye from N = 1 minipig per time point). Images are from MP1, MP3, and MP6-8.

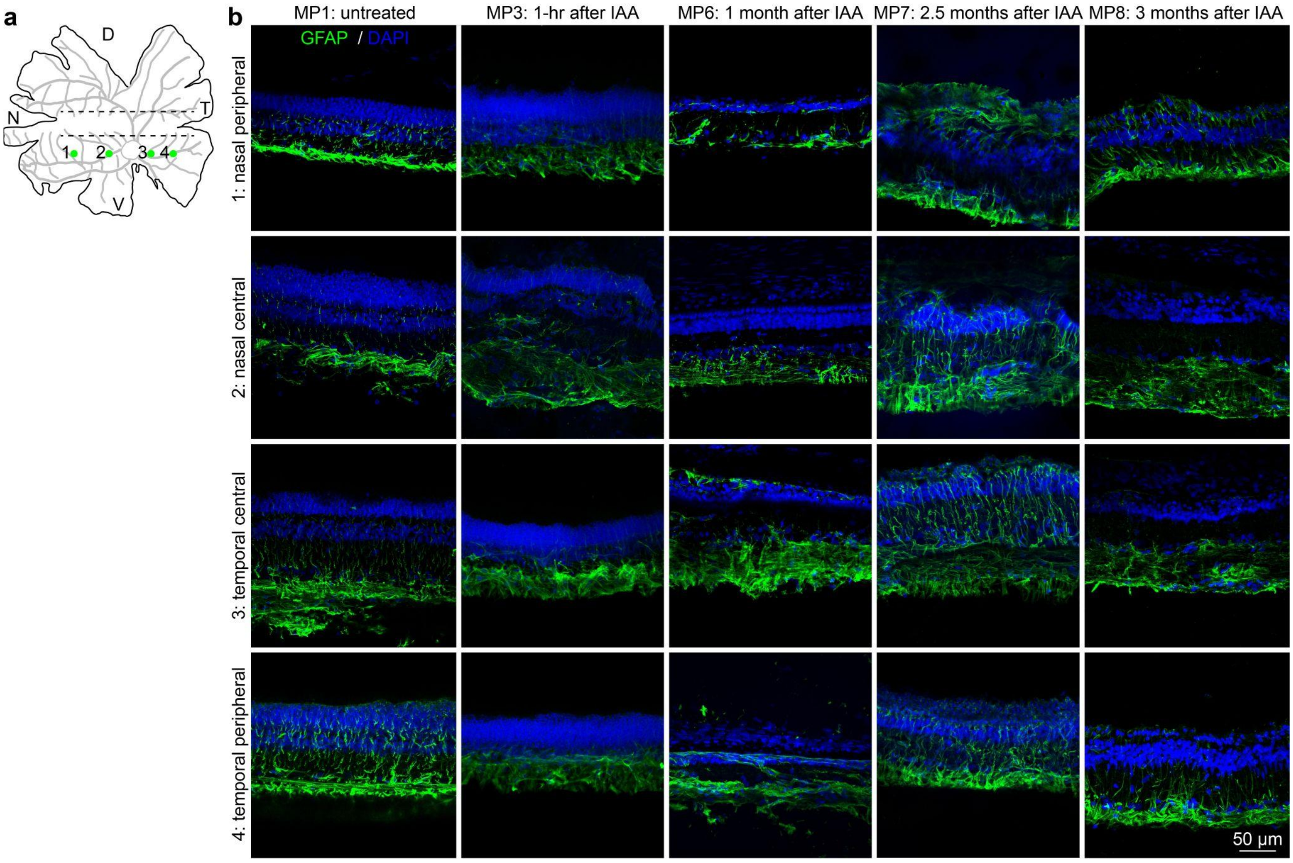

Supplementary Figure 17 | IHC staining against GFAP at the level of the optic disc. **a**, Drawing of a flattened retina. The dashed lines delimit the area centralis, while the green circles indicate the points corresponding to the images in panel **b** (1: nasal peripheral; 2: nasal central; 3: temporal central; 4: temporal peripheral). D: dorsal; T: temporal; V: ventral; N: nasal. **b**, Images of the stained retinas at various time points (untreated control, 1 hour after IAA administration, 1 month after IAA administration, 2.5 months and 4 months after IAA administration) and locations. The images at the same time point are from the same eye. The images at different time points are from different animals (n = 1 eye from N = 1 minipig per time point). Images are from MP1, MP3, and MP6-8.

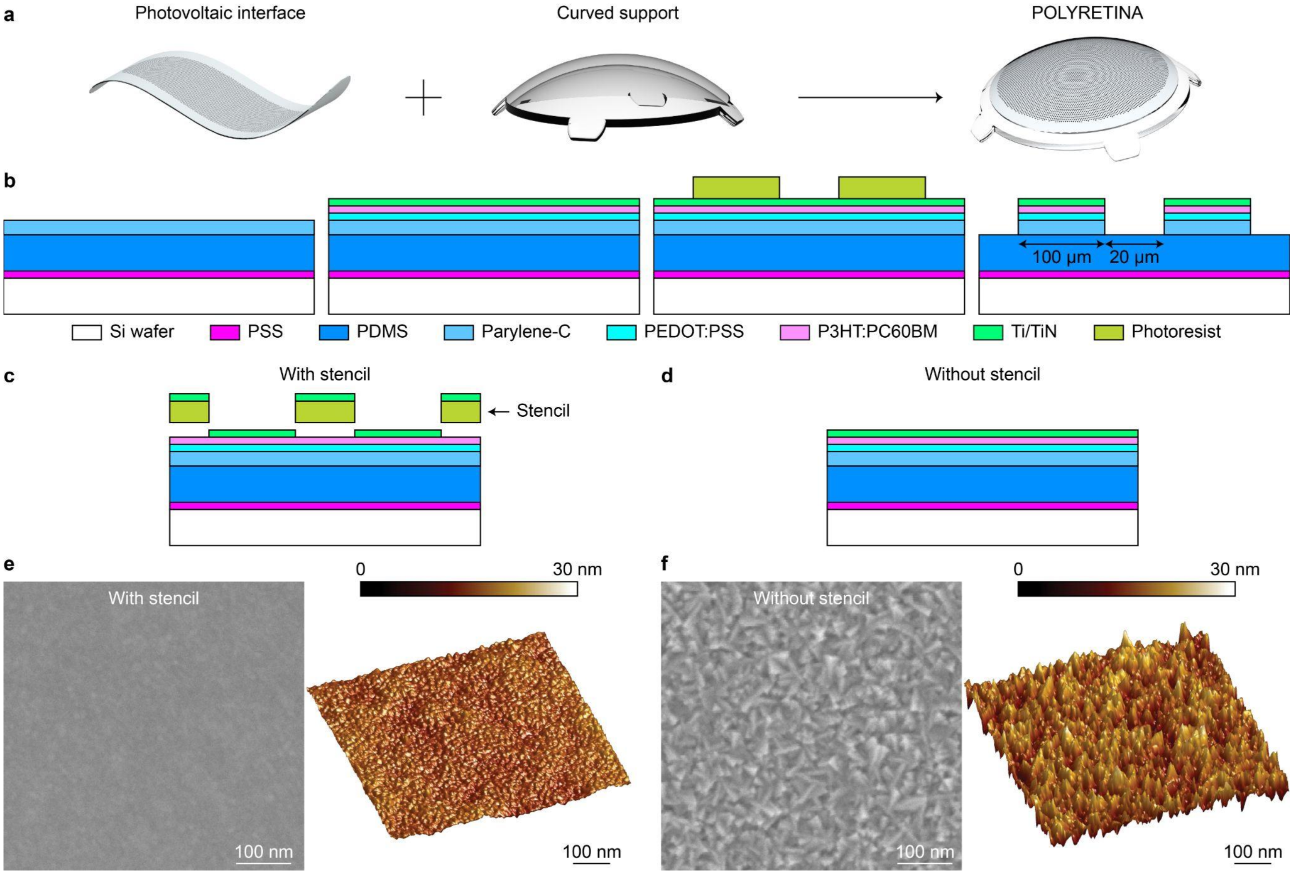

Supplementary Figure 18 | Manufacturing and characterisation of the photovoltaic pixels. **a**, POLYRETINA is manufactured by bonding a photovoltaic interface onto a curved PDMS dome. **b**, Main microfabrication steps for producing the photovoltaic pixels deposited directly onto a 5-µm thick layer of parylene-C and patterned via photolithography. PSS, PDMS, PEDOT:PSS, and P3HT:PC60BM are deposited by spin-coating; parylene-C is deposited by chemical vapour deposition at room temperature; Ti (100 nm) and TiN (100 nm) are deposited by magnetron sputtering. **c,d**, Deposition strategy for the Ti/TiN cathodes through a stencil mask (**c**) or directly onto the wafer (**d**). **e,f**, Scanning electron microscope and AFM images of the Ti/TiN surface sputtered through a stencil mask (**e**) or directly onto the wafer (**f**). The colour bars with the atomic force microscope images show the surface roughness. Representative example from 3 independent replicates.

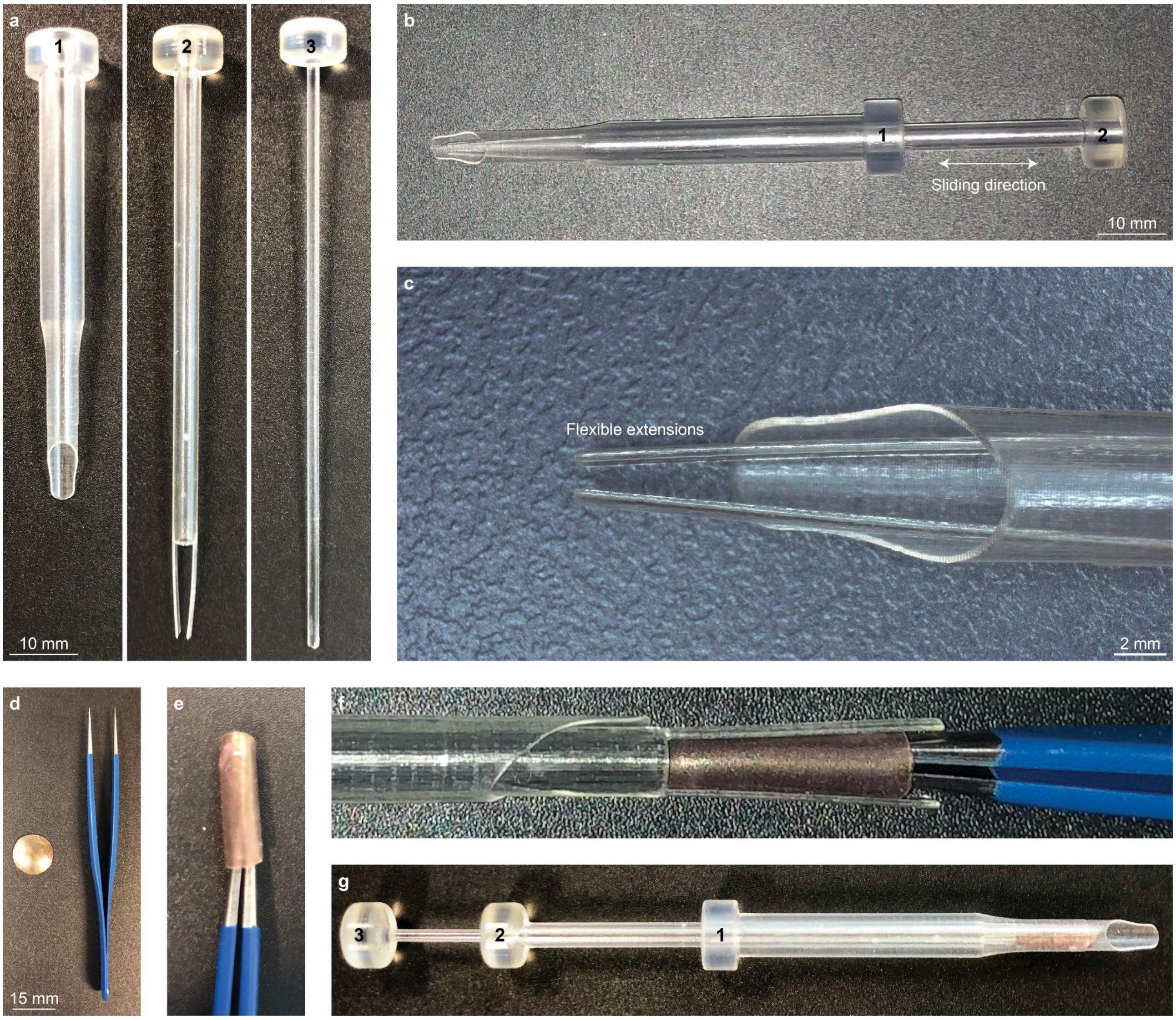

Supplementary Figure 19 | Injector. **a**, Pictures of the three components of the injector: (1) a bevelled tube of 4 mm in external diameter, (2) a narrow tube with thin and flexible extensions and (3) a plunger. **b**, First phase of the assembly, in which the narrow tube with thin and flexible extensions is inserted into the bevelled tube. **c**, Magnification of the tip of the injector showing the flexible extensions. **d**, POLYRETINA ready to be rolled with a tweezer. **e**, POLYRETINA rolled and held by the tweezer. **f**, Insertion of the rolled POLYRETINA into the flexible extensions. **g**, POLYRETINA loaded into the injector with the plunger inserted.
