# Supplementary material for: POLYRETINA restores light responses in vivo in blind Göttingen minipigs
Source: Nat Commun. 2022 Jun 27;13:3678. doi: 10.1038/s41467-022-31180-z (PMC9237028; doi:10.1038/s41467-022-31180-z)
Supplement: Supplementary file 3 — Description of Additional Supplementary Information [file 41467_2022_31180_MOESM3_ESM.pdf]

Description of Additional Supplementary Information

Title: Supplementary Movie 1

Description: POLYRETINA injection in blind Göttingen minipigs.
