# Supplementary material for: POLYRETINA restores light responses in vivo in blind Göttingen minipigs
Source: Nat Commun. 2022 Jun 27;13:3678. doi: 10.1038/s41467-022-31180-z (PMC9237028; doi:10.1038/s41467-022-31180-z)
Supplement: Supplementary file 5 — Reporting Summary [file 41467_2022_31180_MOESM5_ESM.pdf]

nature portfolio

Corresponding author(s): Diego Ghezzi

Last updated by author(s): Apr 26, 2022

Reporting Summary

Nature Portfolio wishes to improve the reproducibility of the work that we publish. This form provides structure for consistency and transparency in reporting. For further information on Nature Portfolio policies, see our Editorial Policies and the Editorial Policy Checklist.

Statistics

For all statistical analyses, confirm that the following items are present in the figure legend, table legend, main text, or Methods section.

n/a	Confirmed	
☐	☒	The exact sample size (n) for each experimental group/condition, given as a discrete number and unit of measurement
☐	☒	A statement on whether measurements were taken from distinct samples or whether the same sample was measured repeatedly
☐	☒	The statistical test(s) used AND whether they are one- or two-sided<br>*Only common tests should be described solely by name; describe more complex techniques in the Methods section.*
☒	☐	A description of all covariates tested
☐	☒	A description of any assumptions or corrections, such as tests of normality and adjustment for multiple comparisons
☐	☒	A full description of the statistical parameters including central tendency (e.g. means) or other basic estimates (e.g. regression coefficient) AND variation (e.g. standard deviation) or associated estimates of uncertainty (e.g. confidence intervals)
☐	☒	For null hypothesis testing, the test statistic (e.g. F, t, r) with confidence intervals, effect sizes, degrees of freedom and P value noted<br>*Give P values as exact values whenever suitable.*
☒	☐	For Bayesian analysis, information on the choice of priors and Markov chain Monte Carlo settings
☒	☐	For hierarchical and complex designs, identification of the appropriate level for tests and full reporting of outcomes
☒	☐	Estimates of effect sizes (e.g. Cohen's d, Pearson's r), indicating how they were calculated

Our web collection on statistics for biologists contains articles on many of the points above.

Software and code

Policy information about availability of computer code

Data collection	SD-OCT was performed using an SD-OCT system (Envisu TM R2210 VHR; Bioptigen) with its proprietary software (InVivoVue, version 2.4.340; Bioptigen). Electrophysiology data were acquired with WinAver (version 1.12; Biomedica Mangoni). IHC samples were imaged using a confocal microscope (LSM880, Zeiss) with its proprietary software (Zen, black edition; Zeiss). H&E images were acquired using a slide scanner microscope (VS120; Olympus) with its proprietary software (VS-ASW; Olympus). POLYRETINA transmittance was measured with a power meter (PD300-R Juno; Ophir Optronics Solutions). AFM images were obtained with a Bruker Dimension icon microscope. Surgical procedures were recorded with a camera system (EvoHD; Leica Microsystems).
Data analysis	SD-OCT images were processed in ImageJ (Fiji, version 1.53m). Electrophysiology data were analysed in WinAver (version 1.12; Biomedica Mangoni) and MATLAB (version 2020a; MathWorks). Histological images were analysed in ImageJ (Fiji, version 1.53m). The threshold was set using the Otsu method available in ImageJ. AFM images were analysed in NanoScope (version 9.4; Bruker). Surgical videos were edited in iMovie (version 10.3.2; Apple). Statistical analysis and graphical representation were performed with Prism (version 9.3.1; GraphPad Software). Power analysis was performed with G*Power (version 3.1.9.5).

For manuscripts utilizing custom algorithms or software that are central to the research but not yet described in published literature, software must be made available to editors and reviewers. We strongly encourage code deposition in a community repository (e.g. GitHub). See the Nature Portfolio guidelines for submitting code & software for further information.

Data

Policy information about availability of data

All manuscripts must include a data availability statement. This statement should provide the following information, where applicable:

- Accession codes, unique identifiers, or web links for publicly available datasets
- A description of any restrictions on data availability
- For clinical datasets or third party data, please ensure that the statement adheres to our policy

The authors declare that the data supporting the findings of this study are available in the paper and its supplementary information files. Source data are provided with this paper.

Field-specific reporting

Please select the one below that is the best fit for your research. If you are not sure, read the appropriate sections before making your selection.

[x] Life sciences [ ] Behavioural & social sciences [ ] Ecological, evolutionary & environmental sciences

For a reference copy of the document with all sections, see nature.com/documents/nr-reporting-summary-flat.pdf

Life sciences study design

All studies must disclose on these points even when the disclosure is negative.

Sample size	The sample size was determined via power analysis performed with G*Power.
Data exclusions	No data were excluded
Replication	Post-mortem histological assays have been performed at various time points (n = 1 eye from N = 1 minipig per time point) and at eight locations corresponding to the peripheral nasal retina, the central nasal retina, the central temporal retina, and the peripheral temporal retina at the level of either the area centralis or the optic disc.<br>Optical transparency of POLYRETINA was measured on two samples.<br>All the other experiments were replicated at least in triplicate.<br>All attempts were successful. Not all minipigs injected with IAA were included in the experiment with POLYRETINA (Figs. 5-8). Some of these animals were used to practice and optimise the surgery, the injector and the retinal tacks. We still included the data from these animals in the first characterisation of the IAA-treatment (Figs. 1-3 and related supplementary figures) but not in the recovery study with POLYRETINA (Figs. 5-8).
Randomization	In the study, there are two groups of animals. Blind minipigs (9 animals) and controls (2 animals). Allocation was random.
Blinding	Investigators were not blinded to group allocation. The effect of IAA (blind minipigs) was immediately visible from SD-OCT scans and in-vivo recordings. Since there were 9 blind minipigs and 2 controls over 3 years, the experimenters could easily recognise them.

Reporting for specific materials, systems and methods

We require information from authors about some types of materials, experimental systems and methods used in many studies. Here, indicate whether each material, system or method listed is relevant to your study. If you are not sure if a list item applies to your research, read the appropriate section before selecting a response.

Materials & experimental systems

n/a	Involved in the study	
[ ]	[x]	Antibodies
[x]	[ ]	Eukaryotic cell lines
[x]	[ ]	Palaeontology and archaeology
[ ]	[x]	Animals and other organisms
[x]	[ ]	Human research participants
[x]	[ ]	Clinical data
[x]	[ ]	Dual use research of concern

Methods

n/a	Involved in the study	
[x]	[ ]	ChIP-seq
[x]	[ ]	Flow cytometry
[x]	[ ]	MRI-based neuroimaging

Antibodies

Antibodies used	List of primary antibodies with catalog number, supplier and concentration: anti-rhodopsin, Abcam AB5417, 1:300; anti-L/Mopsin, Abcam AB5405, 1:500; anti-S opsin, Merck AB5407, 1:500; anti-Na+/K+ATPase (α3 Subunit), Sigma A273, 1:500; anti-Iba1, Wako 019-19741, 1:500; anti-GFAP, Dako Z0334, 1:1000.

	List of secondary antibodies with catalog number, supplier and concentration: anti-mouse AlexaFluor 488, 1:500, A11001, Thermofisher; anti-rabbit AlexaFluor 488, 1:500, SAB4600044, Sigma.
Validation	Concentrations for anti-rhodopsin and anti-L/M opsin were obtained from the following publication on swine: Investigative Ophthalmology & Visual Science October 2011, Vol.52, 7917-7923 (doi:https://doi.org/10.1167/iovs.11-7849). anti-GFAP and anti-Iba1 antibodies are frequently in use in our laboratory and the concentrations we were using in mice tissues were working on minipig retinas too. For anti-S opsin and anti-Na+/K+ATPase, we performed tests to validate their best concentrations. The tests were done on 30um sections from a minipig's eye, and the following concentrations were tested: 1:250 / 1:500 and 1:1000. The best concentration for both antibodies was identified to be 1:500.

Animals and other organisms

Policy information about studies involving animals; ARRIVE guidelines recommended for reporting animal research

Laboratory animals	One-year-old female Göttingen minipigs obtained from Ellegaard Göttingen Minipigs
Wild animals	No wild animals were used in the study
Field-collected samples	No field-collected samples were used in the study
Ethics oversight	Experiments were approved by the Département de l'emploi, des affaires sociales et de la santé (DEAS), Direction générale de la santé de la République et Canton de Genève in Switzerland (authorization number GE/120/19).

Note that full information on the approval of the study protocol must also be provided in the manuscript.
